# Supplementary material for: Deletion of wfs1 Impairs Oligodendrocyte Precursor Cells Dorsal Distribution and Myelination Through the wfs1-hmgcs1 Axis in Zebrafish
Source: Biology (Basel). 2026 Aug 16;15(16):1402. doi: 10.3390/biology15161402 (PMC13510151; doi:10.3390/biology15161402)
Supplement: Supplementary file 1 [file biology-15-01402-s001.zip › File S1-Transcriptome sequencing data and differentially expressed genes (DEGs) in wfs1-- zebrafish at 5 dpf/2.KEGG/wfs1KOvsWT/GSEA/wfs1KO/CARBON_METABOLISM/CARBON_METABOLISM(DRE01200).html]

Details for gene set CARBON\_METABOLISM(DRE01200)[GSEA]

|  || Dataset | deg.sample |
| Phenotype | sample.cls |
| Upregulated in class | wfs1KO |
| GeneSet | CARBON\_METABOLISM(DRE01200) |
| Enrichment Score (ES) | 0.34956396 |
| Normalized Enrichment Score (NES) | 1.7086661 |
| Nominal p-value | 0.0 |
| FDR q-value | 0.09300245 |
| FWER p-Value | 0.191 |
Table: GSEA Results Summary

  

Fig 1: Enrichment plot: CARBON\_METABOLISM(DRE01200)      
 Profile of the Running ES Score & Positions of GeneSet Members on the Rank Ordered List

  

| PROBE | DESCRIPTION (from dataset) | GENE SYMBOL | GENE\_TITLE | RANK IN GENE LIST | RANK METRIC SCORE | RUNNING ES | CORE ENRICHMENT || 1 | 541329 | gcsha |  |  | 17 | 4.955 | 0.0315 | Yes |
| 2 | 560753\_1 | tpi1b |  |  | 49 | 4.890 | 0.0621 | Yes |
| 3 | 406762\_1 | pgd |  |  | 66 | 4.835 | 0.0928 | Yes |
| 4 | 116517 | adh5 |  |  | 148 | 4.502 | 0.1193 | Yes |
| 5 | 445233 | me2 |  |  | 202 | 4.186 | 0.1446 | Yes |
| 6 | 335715 | mdh1ab |  |  | 215 | 4.103 | 0.1707 | Yes |
| 7 | 560827\_1 | prps1b |  |  | 229 | 4.021 | 0.1963 | Yes |
| 8 | 437019\_1 | pcca |  |  | 307 | 3.602 | 0.2171 | Yes |
| 9 | 403070\_1 | rgn |  |  | 363 | 3.424 | 0.2375 | Yes |
| 10 | 561416\_1 | pfkpb |  |  | 497 | 2.998 | 0.2527 | Yes |
| 11 | 406702 | pdha1a |  |  | 691 | 2.614 | 0.2635 | Yes |
| 12 | 570579\_1 | g6pd |  |  | 730 | 2.516 | 0.2785 | Yes |
| 13 | 317746 | suclg2 |  |  | 871 | 2.285 | 0.2888 | Yes |
| 14 | 100006589\_1 | idh1 |  |  | 876 | 2.275 | 0.3034 | Yes |
| 15 | 550579\_1 | idh3g |  |  | 1275 | 1.830 | 0.3027 | Yes |
| 16 | 58068 | pcxb |  |  | 1914 | 1.406 | 0.2916 | Yes |
| 17 | 445129\_1 | sdhc |  |  | 1953 | 1.389 | 0.2994 | Yes |
| 18 | 406688\_1 | got2a |  |  | 2167 | 1.300 | 0.3011 | Yes |
| 19 | 563642 | sdsl |  |  | 2618 | 1.139 | 0.2942 | Yes |
| 20 | 321224 | hkdc1 |  |  | 2737 | 1.100 | 0.2976 | Yes |
| 21 | 407673\_1 | rpia |  |  | 2807 | 1.082 | 0.3024 | Yes |
| 22 | 114551 | pklr |  |  | 2861 | 1.068 | 0.3077 | Yes |
| 23 | 368262\_1 | dlst |  |  | 2921 | 1.056 | 0.3126 | Yes |
| 24 | 101886255 | LOC101886255 |  |  | 2978 | 1.042 | 0.3176 | Yes |
| 25 | 436762 | sdhda |  |  | 3054 | 1.024 | 0.3218 | Yes |
| 26 | 322670 | aco2 |  |  | 3094 | 1.015 | 0.3272 | Yes |
| 27 | 436914 | acss2 |  |  | 3398 | 0.954 | 0.3238 | Yes |
| 28 | 553804 | mcee |  |  | 3424 | 0.947 | 0.3291 | Yes |
| 29 | 321224\_1 | hkdc1 |  |  | 3521 | 0.925 | 0.3320 | Yes |
| 30 | 792824 | pgp |  |  | 3560 | 0.918 | 0.3368 | Yes |
| 31 | 751668 | gck |  |  | 3655 | 0.901 | 0.3396 | Yes |
| 32 | 445288 | acads |  |  | 3897 | 0.848 | 0.3375 | Yes |
| 33 | 406696 | pgk1 |  |  | 3987 | 0.833 | 0.3401 | Yes |
| 34 | 30643 | acat2 |  |  | 4174 | 0.798 | 0.3394 | Yes |
| 35 | 436672 | pdha1b |  |  | 4206 | 0.792 | 0.3435 | Yes |
| 36 | 793926 | tkfc |  |  | 4368 | 0.766 | 0.3434 | Yes |
| 37 | 30068 | cat |  |  | 4462 | 0.750 | 0.3453 | Yes |
| 38 | 560944 | pfkpa |  |  | 4480 | 0.748 | 0.3496 | Yes |
| 39 | 568001\_3 | pfkmb |  |  | 4822 | 0.702 | 0.3433 | No |
| 40 | 562149 | sdhb |  |  | 5566 | 0.630 | 0.3240 | No |
| 41 | 317746\_1 | suclg2 |  |  | 5836 | 0.604 | 0.3194 | No |
| 42 | 799963 | gpt2 |  |  | 6058 | 0.581 | 0.3162 | No |
| 43 | 449662 | acox1 |  |  | 6280 | 0.559 | 0.3128 | No |
| 44 | 570579\_2 | g6pd |  |  | 6769 | 0.513 | 0.3008 | No |
| 45 | 317737\_1 | glud1a |  |  | 6910 | 0.499 | 0.2996 | No |
| 46 | 282672 | fbp1b |  |  | 7040 | 0.488 | 0.2987 | No |
| 47 | 436603 | agxta |  |  | 7216 | 0.472 | 0.2962 | No |
| 48 | 570579 | g6pd |  |  | 7224 | 0.472 | 0.2990 | No |
| 49 | 402939 | zgc:77938 |  |  | 7452 | 0.457 | 0.2948 | No |
| 50 | 436672\_1 | pdha1b |  |  | 8125 | 0.417 | 0.2763 | No |
| 51 | 140620 | prps1a |  |  | 8296 | 0.413 | 0.2736 | No |
| 52 | 406330 | got1 |  |  | 8454 | 0.406 | 0.2713 | No |
| 53 | 323107 | pgam1a |  |  | 8482 | 0.404 | 0.2730 | No |
| 54 | 100006589 | idh1 |  |  | 8531 | 0.401 | 0.2741 | No |
| 55 | 415247\_1 | idh3b |  |  | 8869 | 0.377 | 0.2659 | No |
| 56 | 558487 | eno4 |  |  | 8873 | 0.376 | 0.2683 | No |
| 57 | 322482 | adh8a |  |  | 8920 | 0.373 | 0.2692 | No |
| 58 | 402827 | hao1 |  |  | 9271 | 0.348 | 0.2604 | No |
| 59 | 436647 | aldh6a1 |  |  | 9402 | 0.340 | 0.2585 | No |
| 60 | 335817 | pkma |  |  | 9917 | 0.309 | 0.2443 | No |
| 61 | 378713 | tktb |  |  | 10030 | 0.301 | 0.2427 | No |
| 62 | 768196 | me1 |  |  | 10781 | 0.256 | 0.2207 | No |
| 63 | 393938 | fh |  |  | 10925 | 0.248 | 0.2178 | No |
| 64 | 406791\_2 | hk1 |  |  | 11184 | 0.234 | 0.2112 | No |
| 65 | 403086 | gpt2l |  |  | 11238 | 0.231 | 0.2110 | No |
| 66 | 402841\_1 | adh8b |  |  | 11968 | 0.193 | 0.1893 | No |
| 67 | 437019 | pcca |  |  | 12058 | 0.187 | 0.1877 | No |
| 68 | 406421 | acox3 |  |  | 12372 | 0.170 | 0.1789 | No |
| 69 | 541435\_1 | acss1 |  |  | 12397 | 0.169 | 0.1793 | No |
| 70 | 406339 | hk2 |  |  | 12475 | 0.165 | 0.1779 | No |
| 71 | 566398 | adpgk2 |  |  | 12909 | 0.145 | 0.1652 | No |
| 72 | 192309 | tpi1a |  |  | 12926 | 0.144 | 0.1656 | No |
| 73 | 324556 | taldo1 |  |  | 13048 | 0.137 | 0.1627 | No |
| 74 | 791730 | got1l1 |  |  | 13108 | 0.135 | 0.1617 | No |
| 75 | 101882140 | LOC101882140 |  |  | 13313 | 0.125 | 0.1561 | No |
| 76 | 317743 | gapdh |  |  | 13695 | 0.107 | 0.1448 | No |
| 77 | 246094 | gpia |  |  | 13750 | 0.104 | 0.1437 | No |
| 78 | 406330\_1 | got1 |  |  | 14060 | 0.089 | 0.1346 | No |
| 79 | 559207 | ogdhl |  |  | 14255 | 0.080 | 0.1290 | No |
| 80 | 406791 | hk1 |  |  | 14413 | 0.073 | 0.1245 | No |
| 81 | 322339 | cs |  |  | 15243 | 0.035 | 0.0986 | No |
| 82 | 557919\_1 | adpgk |  |  | 15264 | 0.034 | 0.0982 | No |
| 83 | 406299 | sucla2 |  |  | 15431 | 0.025 | 0.0931 | No |
| 84 | 282672\_1 | fbp1b |  |  | 15912 | 0.002 | 0.0780 | No |
| 85 | 334116\_1 | eno1a |  |  | 16552 | -0.019 | 0.0579 | No |
| 86 | 334116 | eno1a |  |  | 16760 | -0.025 | 0.0516 | No |
| 87 | 402841 | adh8b |  |  | 16797 | -0.026 | 0.0506 | No |
| 88 | 569348\_1 | h6pd |  |  | 17148 | -0.038 | 0.0398 | No |
| 89 | 405861 | pccb |  |  | 17313 | -0.046 | 0.0350 | No |
| 90 | 797715 | ogdhb |  |  | 17610 | -0.059 | 0.0260 | No |
| 91 | 570678 | glyctk |  |  | 17707 | -0.063 | 0.0234 | No |
| 92 | 100144628 | shmt2 |  |  | 17906 | -0.072 | 0.0176 | No |
| 93 | 368912 | echs1 |  |  | 18310 | -0.089 | 0.0055 | No |
| 94 | 335974 | got2b |  |  | 18318 | -0.090 | 0.0058 | No |
| 95 | 334897\_1 | rpe |  |  | 18449 | -0.096 | 0.0024 | No |
| 96 | 553283 | pcxa |  |  | 18484 | -0.098 | 0.0019 | No |
| 97 | 399662 | mdh1aa |  |  | 19301 | -0.135 | -0.0229 | No |
| 98 | 100006589\_2 | idh1 |  |  | 19353 | -0.138 | -0.0237 | No |
| 99 | 550259 | pfklb |  |  | 19460 | -0.142 | -0.0261 | No |
| 100 | 406496 | aldoab |  |  | 19616 | -0.149 | -0.0300 | No |
| 101 | 324201 | dlat |  |  | 19665 | -0.151 | -0.0305 | No |
| 102 | 321928 | phgdh |  |  | 19781 | -0.156 | -0.0332 | No |
| 103 | 792692 | aldoca |  |  | 19868 | -0.160 | -0.0348 | No |
| 104 | 436852 | gcshb |  |  | 20182 | -0.174 | -0.0436 | No |
| 105 | 321621 | gldc |  |  | 20262 | -0.177 | -0.0449 | No |
| 106 | 606663 | psph |  |  | 20450 | -0.185 | -0.0496 | No |
| 107 | 406405 | mdh2 |  |  | 20457 | -0.185 | -0.0486 | No |
| 108 | 406428 | pdhb |  |  | 20690 | -0.198 | -0.0547 | No |
| 109 | 373092 | glud1b |  |  | 20882 | -0.209 | -0.0593 | No |
| 110 | 369193 | aldocb |  |  | 21049 | -0.218 | -0.0632 | No |
| 111 | 394021 | shmt1 |  |  | 21319 | -0.232 | -0.0701 | No |
| 112 | 445290 | acat1 |  |  | 21467 | -0.239 | -0.0732 | No |
| 113 | 450000\_1 | amt |  |  | 21766 | -0.255 | -0.0810 | No |
| 114 | 436850 | suclg1 |  |  | 22189 | -0.278 | -0.0925 | No |
| 115 | 450000 | amt |  |  | 22218 | -0.279 | -0.0916 | No |
| 116 | 569581 | mut |  |  | 22328 | -0.286 | -0.0932 | No |
| 117 | 327165 | pgam1b |  |  | 22471 | -0.294 | -0.0957 | No |
| 118 | 541435 | acss1 |  |  | 22524 | -0.297 | -0.0955 | No |
| 119 | 393455\_1 | hao2 |  |  | 22551 | -0.298 | -0.0944 | No |
| 120 | 564552 | ogdha |  |  | 22588 | -0.301 | -0.0935 | No |
| 121 | 393884 | sdha |  |  | 22785 | -0.312 | -0.0977 | No |
| 122 | 406762 | pgd |  |  | 22903 | -0.320 | -0.0993 | No |
| 123 | 445494 | pgls |  |  | 23031 | -0.330 | -0.1012 | No |
| 124 | 492719 | me3 |  |  | 23069 | -0.332 | -0.1002 | No |
| 125 | 378963 | eno3 |  |  | 23157 | -0.338 | -0.1008 | No |
| 126 | 79378 | agxtb |  |  | 23331 | -0.350 | -0.1040 | No |
| 127 | 402874 | eno2 |  |  | 23379 | -0.353 | -0.1032 | No |
| 128 | 445500 | sdhdb |  |  | 23455 | -0.358 | -0.1032 | No |
| 129 | 386951 | idh2 |  |  | 23851 | -0.383 | -0.1132 | No |
| 130 | 550494 | esd |  |  | 23886 | -0.385 | -0.1118 | No |
| 131 | 336425 | aldoaa |  |  | 24256 | -0.411 | -0.1208 | No |
| 132 | 445129\_2 | sdhc |  |  | 24579 | -0.420 | -0.1282 | No |
| 133 | 317737 | glud1a |  |  | 25006 | -0.446 | -0.1388 | No |
| 134 | 399479 | dldh |  |  | 25027 | -0.448 | -0.1365 | No |
| 135 | 246095 | gpib |  |  | 25103 | -0.454 | -0.1360 | No |
| 136 | 445129 | sdhc |  |  | 25446 | -0.479 | -0.1436 | No |
| 137 | 393926 | idh3a |  |  | 25570 | -0.491 | -0.1444 | No |
| 138 | 560020 | acss2l |  |  | 26041 | -0.533 | -0.1557 | No |
| 139 | 570106 | pfkla |  |  | 26195 | -0.547 | -0.1570 | No |
| 140 | 568448 | aco1 |  |  | 26535 | -0.582 | -0.1640 | No |
| 141 | 798364 | hibch |  |  | 27149 | -0.642 | -0.1791 | No |
| 142 | 100331665 | LOC100331665 |  |  | 27187 | -0.644 | -0.1761 | No |
| 143 | 317746\_2 | suclg2 |  |  | 27320 | -0.651 | -0.1761 | No |
| 144 | 415247 | idh3b |  |  | 27612 | -0.688 | -0.1808 | No |
| 145 | 572733 | pgam2 |  |  | 27679 | -0.698 | -0.1784 | No |
| 146 | 321664 | aldob |  |  | 27744 | -0.709 | -0.1758 | No |
| 147 | 100148522 | si:ch211-217a12.1 |  |  | 27762 | -0.711 | -0.1718 | No |
| 148 | 445505 | fbp2 |  |  | 28134 | -0.761 | -0.1786 | No |
| 149 | 116517\_1 | adh5 |  |  | 28219 | -0.775 | -0.1762 | No |
| 150 | 557518 | tkta |  |  | 28233 | -0.776 | -0.1716 | No |
| 151 | 327512 | psat1 |  |  | 28400 | -0.803 | -0.1717 | No |
| 152 | 447836 | pfkma |  |  | 28916 | -0.902 | -0.1821 | No |
| 153 | 555623 | cps1 |  |  | 28996 | -0.919 | -0.1786 | No |
| 154 | 393668 | eno1b |  |  | 29713 | -1.133 | -0.1939 | No |
| 155 | 335231 | fbp1a |  |  | 29764 | -1.152 | -0.1880 | No |
| 156 | 445094 | pkmb |  |  | 29848 | -1.183 | -0.1830 | No |
| 157 | 560827 | prps1b |  |  | 29931 | -1.217 | -0.1777 | No |
| 158 | 792692\_1 | aldoca |  |  | 30223 | -1.351 | -0.1782 | No |
| 159 | 445233\_1 | me2 |  |  | 30440 | -1.469 | -0.1755 | No |
| 160 | 568001\_1 | pfkmb |  |  | 30537 | -1.531 | -0.1687 | No |
| 161 | 406688 | got2a |  |  | 30634 | -1.617 | -0.1612 | No |
| 162 | 368262 | dlst |  |  | 30863 | -1.822 | -0.1567 | No |
| 163 | 406339\_1 | hk2 |  |  | 31039 | -2.013 | -0.1492 | No |
| 164 | 393427 | zgc:63568 |  |  | 31073 | -2.050 | -0.1370 | No |
| 165 | 403070 | rgn |  |  | 31121 | -2.114 | -0.1248 | No |
| 166 | 407673 | rpia |  |  | 31144 | -2.139 | -0.1117 | No |
| 167 | 560753 | tpi1b |  |  | 31226 | -2.286 | -0.0995 | No |
| 168 | 541329\_1 | gcsha |  |  | 31249 | -2.328 | -0.0852 | No |
| 169 | 792824\_1 | pgp |  |  | 31276 | -2.388 | -0.0706 | No |
| 170 | 561416 | pfkpb |  |  | 31341 | -2.509 | -0.0564 | No |
| 171 | 322670\_1 | aco2 |  |  | 31502 | -2.927 | -0.0425 | No |
| 172 | 568001\_2 | pfkmb |  |  | 31666 | -3.543 | -0.0248 | No |
| 173 | 550579 | idh3g |  |  | 31862 | -4.927 | 0.0009 | No |
Table: GSEA details [plain text format]

  

Fig 2: CARBON\_METABOLISM(DRE01200)      
 Blue-Pink O' Gram in the Space of the Analyzed GeneSet

  

Fig 3: CARBON\_METABOLISM(DRE01200): Random ES distribution      
 Gene set null distribution of ES for **CARBON\_METABOLISM(DRE01200)**
